# Early-onset neonatal sepsis and neonatal antibiotic exposure in Hungary a nationwide population-based study

**DOI:** 10.1007/s00431-026-07455-8

**Published:** 2026-10-02

**Authors:** Judit Mari, Claus Klingenberg, Flavia Rosa-Mangeret, Laura Sára Mlinarics, Miklós Szabó, Andrea Valek, Judit Klára Kiss, Gyula Tálosi

**Affiliations:** 1 Department of Pediatrics, University of Szeged, Szeged, Hungary; 2 Research Group for Child and Adolescent Health, Faculty of Health Sciences, UiT-The Arctic University of Norway, Tromsø, Norway; 3 Department of Pediatrics and Adolescence Medicine, University Hospital of North Norway, Tromsø, Norway; 4 Neonatology and Paediatric Intensive Care Unit, Geneva University Hospitals and Geneva University, Geneva, Switzerland; 5 Department of Neonatology, Centre of Pediatrics, Semmelweis University, Budapest, Hungary; 6 Heim Pál National Institute of Pediatrics, Budapest, Hungary; 7 Bács-Kiskun County Teaching Hospital, Kecskemét, Hungary

**Keywords:** Perinatal epidemiology, Neonatal early-onset sepsis, Antibiotic exposure, Hungary, Central Europe

## Abstract

Neonatal sepsis remains a challenge in perinatal medicine. We aimed to estimate incidence of culture positive early-onset sepsis (CP-EOS), causative pathogens and overall antibiotic exposure in newborn infants in Hungary. Retrospective national registry analysis (2020–2023). Data were mainly collected from the Hungarian National Perinatal Registry covering all level II-III neonatal intensive care units (NICUs) in Hungary. The number of livebirths in each gestational age group was provided by Hungarian Central Statistical Office. Overall CP-EOS incidence was 0.41/1,000 livebirths (0.20/1,000 ≥ 35 weeks gestation, 7.0/1,000 < 35 weeks gestation). Main pathogens were *Escherichia coli* (33.3%) and Group B streptococci -GBS (24.5%). Ten of 49 (20.4%) infants with *E. coli* sepsis died. No GBS related mortality occurred. Lumbar puncture was only performed in 30/147 (20.4%) of the CP-EOS cases. No cases of isolated meningitis occurred in the absence of CP-EOS. Newborns presenting with respiratory distress in the first 72 h were five times more likely to receive antibiotics (OR 4.9 CI 4.6–5.2) than infants without respiratory distress. Overall, 63.9% of NICU-treated neonates received antibiotics for a median (IQR) duration of 5 (3–8) days. A minimum of 3.2% of all neonates in Hungary received antibiotics, acknowledging that some more neonates are treated on postnatal wards and Level I NICUs which are not included in the perinatal registry. A substantial number (15.6%) of blood cultures were taken without subsequent antibiotic commencement.

*Conclusions*: Our results provide the first national incidence estimates for CP-EOS in Central-Eastern Europe. The study identified high antibiotic use with a concurrently relatively low EOS incidence. The results support implementing new strategies to reduce unnecessary antibiotic exposure in the newborn period.

**Table:** 

**What is Known:** • *Neonatal early-onset sepsis (EOS) causes substantial neonatal morbidity, prompting widespread, frequent empirical antibiotic use*. • *Unnecessary early-life antibiotic exposure shifts the infant gut microbiota and increases risks for long-term adverse health outcomes*.	
**What is New:** • *This first nationwide Central-Eastern European study reveals a low EOS incidence (0.41/1,000 live births) but high NICU antibiotic exposure (63.9%) in Hungary*. • *Escherichia coli has overtaken GBS as the leading EOS pathogen, causing significant mortality primarily in preterm infants*.

**Supplementary Information:**

The online version contains supplementary material available at https://doi.org/10.1007/s00431-026-07455-8.

## Introduction

Neonatal sepsis remains one of the most critical challenges in perinatal medicine worldwide. Although it is a relatively rare condition, neonatal sepsis is associated with significant morbidity and mortality, particularly among preterm infants [1–3]. Early detection and targeted treatment are essential, but the diagnosis of neonatal sepsis is often hindered by non-specific clinical signs and the absence of reliable sepsis specific biomarkers [1, 4, 5]. As a result, empirical antibiotic therapy is commonly initiated, sometimes based solely on gestational age or vague clinical symptoms.

This widespread use of antibiotics in neonatology carries well-documented risks. Prolonged or unnecessary antibiotic exposure in early life has been linked to increased rates of both short-term adverse outcomes (e.g. necrotizing enterocolitis, late-onset sepsis and antibiotic resistance development) and long-term adverse outcomes (e.g. allergic diseases, obesity, diabetes and inflammatory bowel disease) [6–8]. Recent international studies emphasize the need for antibiotic stewardship and evidence-based protocols to guide clinical decision-making, especially for vulnerable preterm populations [9, 10].

One such tool for term and late preterm infants is the Kaiser Permanente Neonatal Early-Onset Sepsis (EOS) Calculator, which assists clinicians in risk stratification based on epidemiological background rates and individual risk factors [11–13]. However, for this tool to be reliably adapted to local practice, robust, country-specific incidence data are required. To date, no comprehensive national data on the incidence of EOS have been published for Hungary, and comparable nationwide data are also lacking from other Central-Eastern European countries. Similarly, the extent and variability of empirical antibiotic use across Hungarian Neonatal Intensive Care Units (NICUs) have not been systematically examined.

In this context, we conducted a nationwide analysis of EOS incidence, causative pathogen profiles, and patterns of antibiotic use across Hungarian NICUs. Our primary goal was to present national data on the EOS incidence, which is important if wanting to implement the EOS risk calculator. Additional objectives included describing the current distribution of EOS pathogens and antibiotic use thereby providing reliable data to inform and strengthen antibiotic stewardship strategies for newborn infants in Hungary.

## Methods

### Study design and setting

This is a retrospective population-based study, based on data from two Hungarian nationwide registries. Data were collected from January 1, 2020 through December 31, 2023. From the Hungarian Central Statistical Office (HCSO) we received data on number of live births per gestational age week during the study period. The Hungarian National Perinatal Registry (HNPR) collects data, manually entered on NICU-discharge, on all neonates admitted to all 20 level III and 12 level II NICUs in Hungary. All neonates with EOS are referred to a level III NICU.

### Study variables and definitions

From the HPNR, demographic data included gestational age (GA), birth weight (BW), sex, multiple birth status and mode of delivery. We also obtained data on respiratory distress during first 72 h of life, defined as a presenting symptom assessed by the attending physician regardless of the use of respiratory support. We recorded the proportion of neonates receiving antibiotics, duration of days of antibiotic exposure per patient, and total antibiotic days per 1,000 live births.

We defined the following outcomes:

- Culture positive EOS (CP-EOS): Isolation of a pathogenic organism from the blood within 72 h of birth, with intention to treat with systemic antibiotics for > 5 days or death attributed to infection. Episodes with a positive blood culture, but treated with antibiotics < 6 days were considered as contamination.
- Early-onset meningitis: Isolation of a pathogenic organism from the cerebrospinal fluid (CSF) within 72 h of birth, with intention to treat with systemic antibiotics for > 5 days or death attributed to infection.
- Contaminants: Organisms regarded as contaminants were Diphtheroids, Micrococcus, Propionibacterium, Corynebacterium, Coagulase-negative Staphylococci (CoNS)
- CP-EOS incidence: Blood culture confirmed EOS cases per 1000 live births in the stratified GA group.
- Early-onset meningitis incidence: Positive CSF culture early-onset meningitis cases per 1000 live births in the stratified GA group.
- CP-EOS-related mortality: Deaths that occurred ≤ 28 days of the NICU admission with a positive blood culture in the first 72 h and sepsis was documented as cause of death in the registry.

The primary outcome was the overall CP-EOS incidence, and the incidence in the two subgroups i) late preterm and term neonates (≥ 35 weeks gestation), the population targeted by the Kaiser Permanente EOS calculator and ii) other preterm neonates (< 35 weeks gestation). Secondary outcomes were causative pathogens from blood cultures and antibiotic exposure among newborn infants in Hungary.

### Statistical analysis

Data were extracted and cleaned in Microsoft Excel and analyzed using IBM SPSS Software Statistics (version 32.0). Descriptive statistics were used to report incidence rates, pathogen distributions, contamination rates, and antibiotic exposure. We present mean and standard deviation (SD) or median and interquartile range (IQR), as appropriate. Comparisons were made between GA groups utilizing Chi square test and Mann–Whitney U test. A significance level of *p* < 0.05 was considered statistically significant.

### Ethics

This non-interventional study was approved by the Scientific and Research Ethics Committee of the Medical Research Council of Hungary (ETT TUKEB), reference number BM/10982–3/2025. Presentation follows the Strengthening the Reporting of Observational Studies in Epidemiology for Newborn Infection reporting guidelines (Supplementary Table 1) [14].

## Results

### Background characteristics and EOS incidence

During the 4-year study period 359,093 neonates were born in Hungary with 11,501 (3.2%) neonates born before 35 weeks gestation. Overall, 20,652 (5.8%) were admitted to one of the Hungarian NICUs, and 147 were diagnosed with a CP-EOS. The total CP-EOS incidence, across all GAs during the study period, was 0.41 per 1,000 live births. Among late preterm and term neonates (≥ 35 weeks' gestation) the CP-EOS incidence was 0.20 per 1,000 live births. Among preterm neonates born < 35 weeks' gestation the CP-EOS incidence was 7.0 per 1,000 live births. Table 1 shows background characteristics of the all NICU-admitted infants, and those with no-EOS versus those with EOS. Newborn infants with CP-EOS had lower GA/BW than those without EOS. There were no differences in sex distribution between CP-EOS and no-EOS group. Respiratory distress was recorded more commonly among infants with CP-EOS. All infants with CP-EOS were given antibiotics. However, antibiotics were also administered to 63.6% of all NICU-admitted infants without EOS and for median (IQR) of 5 (3–8) days.

**Table 1:** Background characteristics and comparison of CP-EOS and no EOS group

	All (*n* = 20,652)	No EOS (*n* = 20,505)	CP-EOS (*n* = 147)	*p* value
Gestational age, weeks (mean, SD)	34.7 (4.1)	34.7 (4.1)	33.3 (5.5)	0.001
< 28, n (%)	1381 (6.7)	1355 (6.6)	26 (17.6)	< 0.001
28–31, n (%)	2788 (13.5)	2760 (13.5)	29 (18.9)	
32–36, n (%)	8758 (42.5)	8718 (42.5)	40 (27.0)	
≥ 37, n (%)	7683 (37.3)	7630 (37.2)	53 (35.8)	
Missing data	41 (0.2)	40 (0.2)	1 (0.7)	
Birth weight, g (mean, SD)	2393 (945)	2394 (944.0)	2203 (1093.0)	0.023
Male sex, n (%)	11,377 (55)	11,302 (55)	75 (51)	0.278
Respiratory distress, n (%)	13,376 (65)	13,260 (65)	120 (82)	< 0.001
Umbilical line, n (%) duration, days (mean, SD)	4380 (21) 5.5 (3.0)	4306 (21) 5.4 (2.9)	74 (50) 3.2 (4.4)	< 0.001 0.04
Antibiotics given, n (%)	13,198 (63.9)	13,034 (63.6)	147 (100)	< 0.001
Antibiotic days (median, IQR)	5 (3–8)	5 (3–7)	10 (7–15)	< 0.001

CP-EOS; culture positive early-onset sepsis, *IQR* interquartile range, *SD* standard deviation

### Pathogen spectrum

**Fig. 1:**
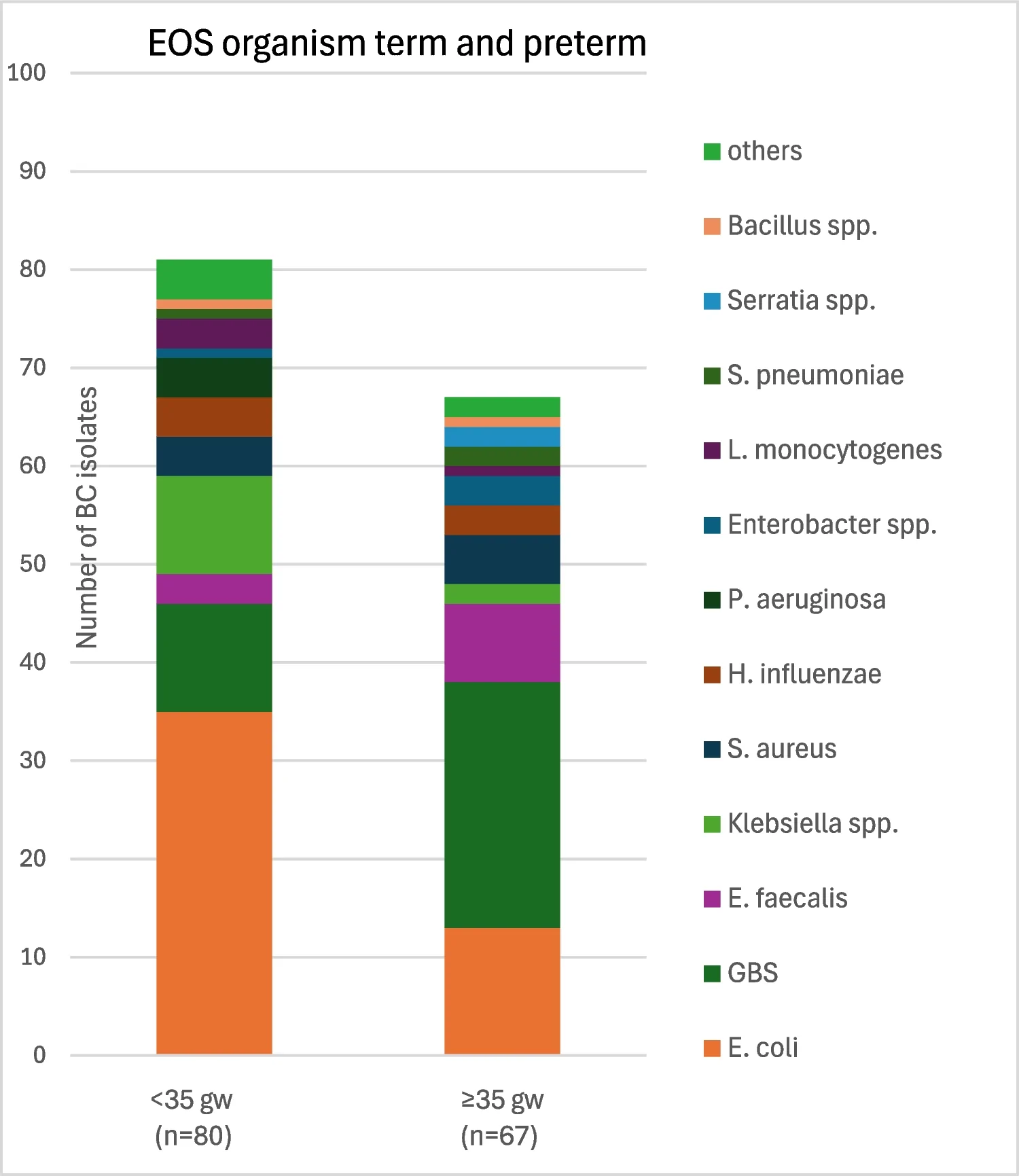
Early-onset sepsis pathogens in both gestational groups

**Fig. 2:**
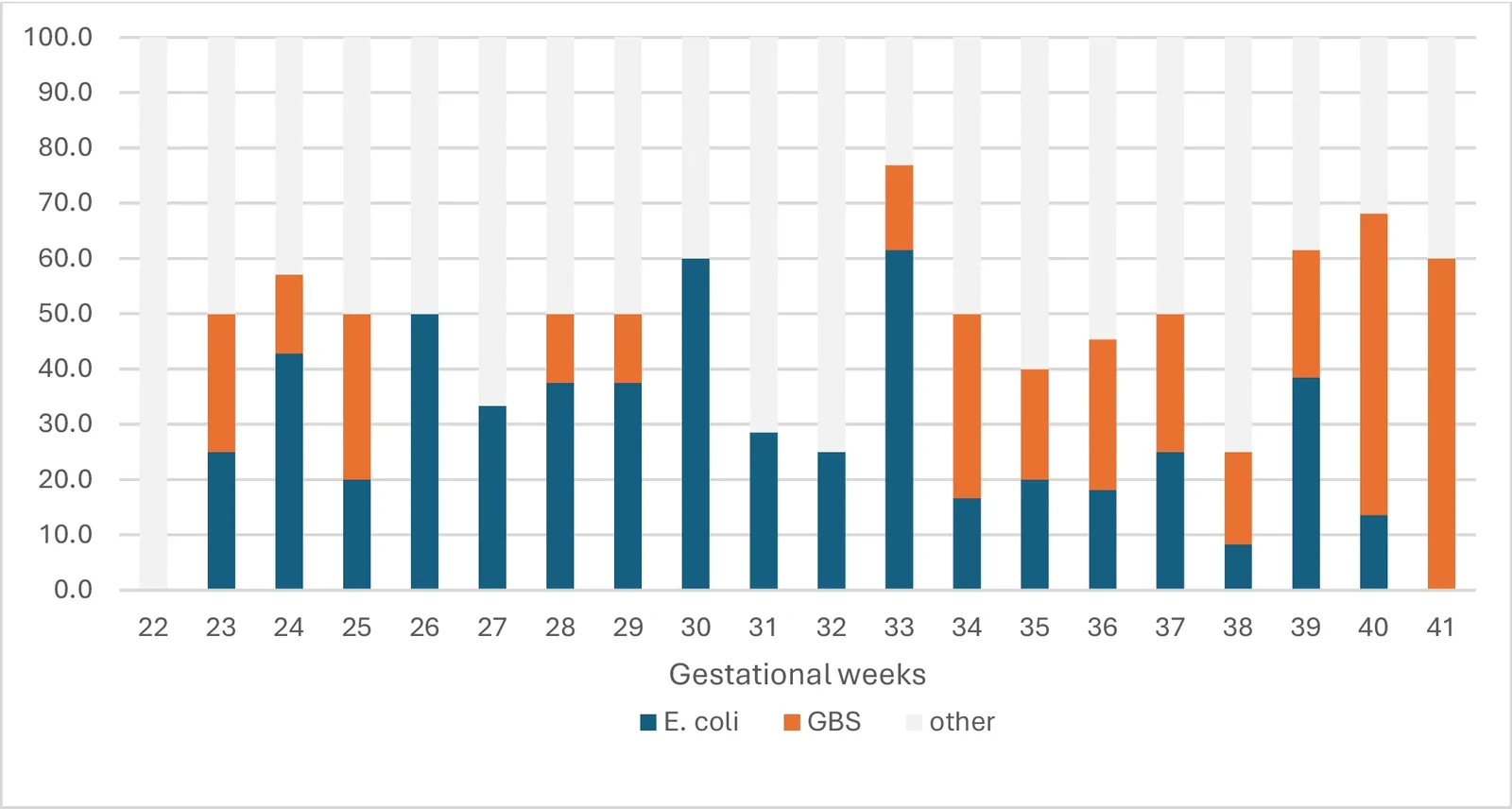
Proportion of *E. coli* and Group B streptococci causing early-onset sepsis across all gestational ages

The most common CP-EOS pathogens were *Escherichia coli* (49/147; 33%) and Group B streptococci (GBS) (36/147; 24.5%) (Fig. 1). GBS was most common among late preterm and term neonates, whereas *E. coli* dominated among preterm infants < 35 weeks gestation (Fig. 2). Other common EOS pathogens were *Enterococcus* spp. (11/147; 7.5%), *Klebsiella* spp. (11/147; 7.5%) and *Staphylococcus aureus* (9/147; 6.1%). We identified four CP-EOS cases (2.7%) caused by *Listeria* spp.

### EOS-related mortality

The overall CP-EOS-related mortality rate was 2.8 per 100,000 live births. Stratified by GA, EOS-related mortality rate was 0.57 per 100,000 for neonates ≥ 35 weeks and 69.6 per 100,000 for those < 35 weeks. The case fatality ratio for EOS was 3.0% (2/67) among term and late preterm neonates; one death was caused by *E. coli* and one by *Serratia marcescens*. Among preterm infants with a gestational age < 35 weeks, the case fatality ratio was 10.0% (8/80); six of the eight deaths were caused by *E. coli* or *Klebsiella* spp.

### Antibiotic use

**Fig. 3:**
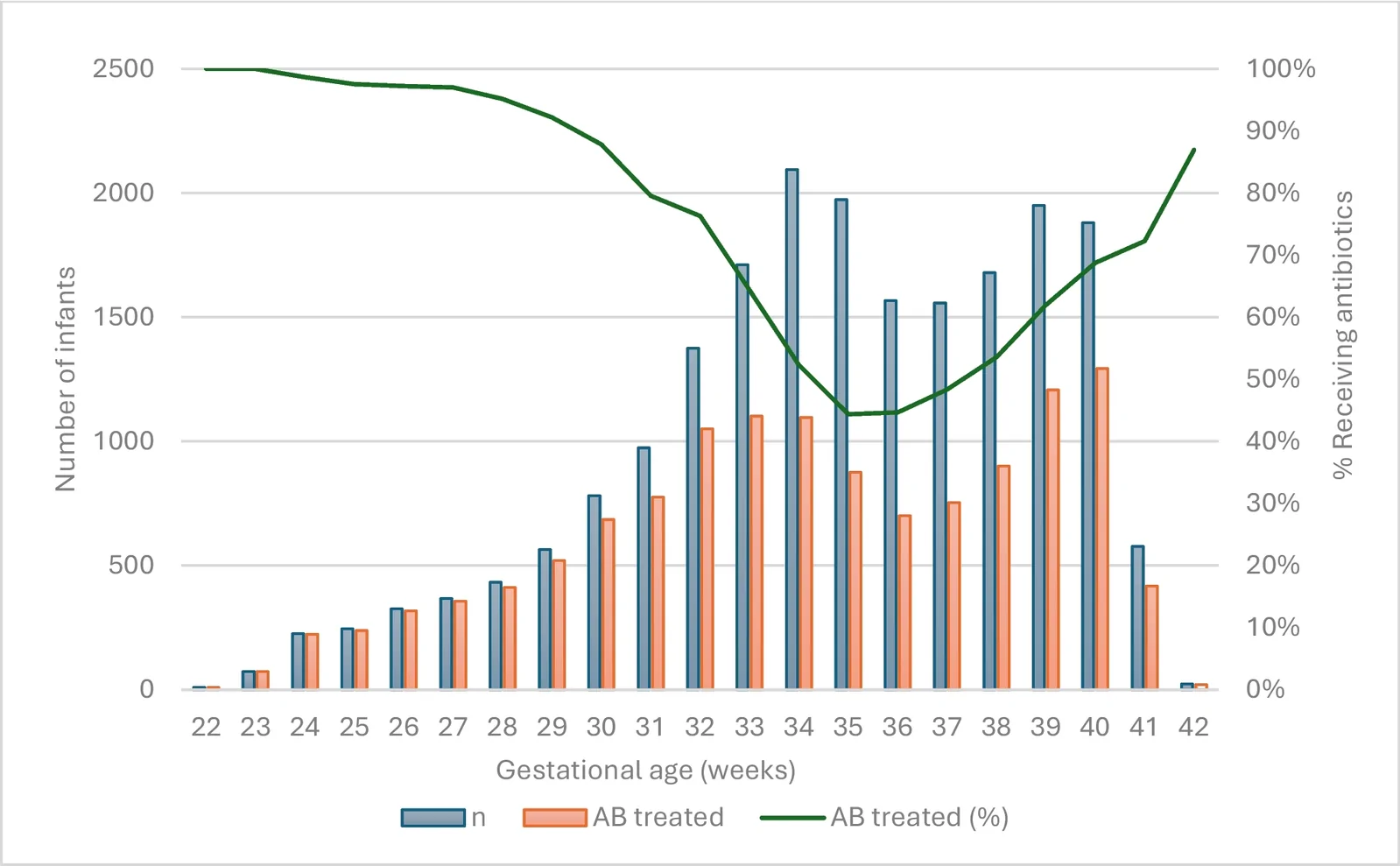
Antibiotic exposure by gestational age among neonates admitted in the NICU. Bars show the total number of infants (blue) and the number who received antibiotic treatment (orange) according to gestational age. The green line represents the proportion of infants receiving antibiotics within each gestational age category

Across all participating NICUs, 63.9% of neonates received antibiotics during their hospitalization. Antibiotic exposure in the NICUs remained stable during the examined 4 years (62.4–65.7%). The proportion of infants who received antibiotics varied widely between different GAs from 44 to 100%, with highest number of treated neonates in the extreme preterm infants (< 28 weeks) and lowest in the late preterm group born between 35–37 weeks (Fig. 3). Newborns with respiratory distress were almost five times more likely to receive antibiotics (OR 4.9 CI 4.6–5.2). Table 2 shows the median (IQR) duration of days of antibiotic exposure per patient treated with antibiotics and total antibiotic days per 1,000 live births, for all infants and the two subgroups. Projecting from the number of neonates receiving antibiotics in the Level II-III NICUs to all livebirths in Hungary during the study period, a minimum of 3.2% (13,198/359,093) of all neonates were treated with antibiotics, and among infants with GA ≥ 35 weeks a minimum of 1.8% (6,203/347,592) were treated. We can only report minium data since we do not have data on the number of neonates treated with antibiotics at postnatal wards and Level I NICUs.

**Table 2:** Summary of results, incidence of culture positive early-onset sepsis (CP-EOS), mortality, and antibiotic use in the different gestational age groups *p < 0.05

	Total	≥ 35 weeks	< 35 weeks
**CP-EOS incidence** (per 1000 live births)	0.41/1,000	0.20/1,000	7.0/1,000 *
**CP-EOS-related mortality rate** (per 100.000 live births)	2.8/100,000	0.57/100,000	69.6/100,000 *
**CP-EOS pathogen distribution**	***E. coli*** (33.3%) GBS (24.5%)	**GBS** (37.3%) *E. coli* (20.9%)	***E. coli*** (43.8%) GBS (13.8%)
**Infants receiving antibiotics**	64% (44–100%)	55% (44–100%)	81% (64–100%)*
**Median (IQR) days of antibiotics in patients treated with antibiotics**	5 (3–8)	5 (3–7)	5 (3–9)
**Total antibiotic days** per 1,000 live births	195/1000	317/1000	178/1000*
**Blood culture contamination**	3.1%	4.3%	2.1%

### Lumbar puncture and meningitis

Lumbar puncture was performed in 20.4% (30/147) of the EOS cases. In the HPRN-database, “clinical meningitis”, without a specified pathogen, was documented in 21 (14.3%) patients. Meningitis confirmed by positive CSF culture was documented in 4 neonates (2.7% of EOS cases), caused by *E. coli* (*n* = 2) and GBS (*n* = 2). In addition, we identified one neonate with neurosyphilis. Meningitis without EOS did not occur in the first 72 h.

### Blood culture indications and contamination rate

During the first 72 h of life, a total of 12,329 blood cultures (59.7% of all admitted neonates) were obtained during the study period. In 1,923 cases, no antibiotic treatment was initiated, indicating that at least 15.6% (1,923/12,329) of blood cultures obtained within the first 72 h of life were taken in infants without sufficient clinical suspicion of EOS to warrant treatment. This occurred more frequently in term and late-preterm infants (gestational age ≥ 35 weeks; 19.4%) than in preterm infants born before 35 weeks (12.3%). Overall, a pathogenic organism was isolated in 147 (1.2%) blood cultures, whereas contaminants were identified in 387 (3.1%).

## Discussion

This population-based study from Hungary provides the first comprehensive report of neonatal culture positive EOS incidence and antibiotic exposure among newborn infants in Eastern Europe. The results reveal important insights on the EOS burden, dominant bacterial pathogens, and current antibiotic exposure.

The EOS incidence in the total Hungarian newborn population was 0.41/1000 live births. EOS was 50 times more common in preterm infants < 35 weeks compared to late preterm/term infants. The EOS incidence in late preterm and term infants (0.2/1,000 live births) in Hungary is low, but comparable to rates reported in other high-income countries [15]. For example, large population-based studies in Norway [16], Sweden [17], and the UK [18] report EOS incidences between 0.2 and 0.9 per 1,000 live births in term or late preterm neonates. A similar EOS incidence has been reported among term infants in North American NICUs, although substantial variation exists between regions and hospitals [19].

Consistent with international data, GBS remains the most common cause of EOS among term infants, whereas *E. coli* predominates in preterm populations [20–22]. However, other pathogens were responsible for nearly half of EOS cases. The overall predominance of *E. coli* is somewhat different from historically observed data previously describing GBS as the leading causative organism [23]. In the last decades an increasing number of *E. coli* EOS cases were reported worldwide, overtaking the declining number of GBS, as the previously leading causative organism. This is thought to be an effect of widespread intrapartum antibiotic prophylaxis use, targeting prevention of GBS-EOS [20]. We observed no GBS-related deaths, and Gram-negative pathogens (predominant *E. coli* and *Klebsiella* spp.) were causative pathogens in nearly all EOS cases who died. Although our study did not capture susceptibility data, data from other countries suggest increasing number of multi-resistant Gram-negative pathogens causing neonatal sepsis [24]. This highlights the need for regular assessment of local antibiotic susceptibility patterns and appropriate revision of national or regional sepsis treatment protocols [24, 25]. It is essential to have country-specific data to inform local risk stratification models and guide the safe and wise use of empirical antibiotics in newborn care.

### Antibiotic exposure

Our findings reveal a high antibiotic use in Hungarian NICUs. We only have data from level II-III NICUs, but we know that many infants also receive antibiotics in level I NICUs and postnatal wards. We found that at least 1.8% of late preterm and term infants are exposed to antibiotics during their stay in the NICU. Nearly two-thirds of all neonates admitted to NICUs received antibiotics, with considerable variation between GAs. This broad range suggests differences in local protocols, risk assessment or diagnostic practices. While prompt empirical treatment is crucial when EOS is suspected, almost 2/3 of all NICU-admitted infants without a documented sepsis episode were also exposed to antibiotics with subsequent risk of developing adverse effects [6–8].

Our data supports the hypothesis that respiratory symptoms might trigger suspicion of EOS and consequent antibiotic prescription in the first 72 h of life as infants with respiratory symptoms were five times more likely to receive antibiotics [26]. However, as respiratory distress is common in many neonatal conditions including respiratory distress syndrome in preterm infants, the specificity of using respiratory distress to guide initiation of antibiotic treatment is low. One study showed that withholding antibiotics in otherwise healthy infants with mild respiratory distress could reduce antibiotic consumption without missing any cases of sepsis [26].

International guidelines and systematic reviews increasingly advocate for targeted antibiotic stewardship programs in NICUs [10]. Studies from the USA and Western Europe demonstrate that local adaptation of validated risk stratification tools, such as serial physical examination and the Kaiser Permanente EOS Calculator, can safely reduce unnecessary antibiotic exposure without increasing missed infections in late preterm and term babies [11–13]. Our data provide local incidence estimates that could enable centres with high antibiotic use to implement the EOS calculator for safer, evidence-based decisions. Moreover, another strategy would be to implement serial physical examination of infants with mild symptoms, which can further reduce antibiotic exposure [27, 28]. A recently updated paediatric sepsis guideline recommends that in children with more vague symptoms of sepsis an observation period of 3 h can be used to decide on either start of antibiotics or whether symptoms most likely are not caused by sepsis and antibiotics can be withheld [29].

### Antibiotic duration in no sepsis cases

A multicentre international study found that antibiotic treatment for suspected EOS is often prolonged beyond 2 days in culture-negative cases, and culture negative EOS was identified as a main driver of antibiotic exposure in early life [30]. In our study median duration of antibiotic therapy in infants with no blood culture proven infection was 5 days. Thus, our data support that antimicrobial stewardship programs in Hungary should focus on shortening antibiotic treatment for culture-negative cases. This has a great potential of reducing antibiotic exposure substantially in the Hungarian setting. The recent Swiss EOS guideline suggests that antibiotic treatment for culture-negative cases needs to be assessed after 24–36 h, and in most cases antibiotics may be stopped at this stage when blood cultures are negative [31].

### Blood culture sampling and contamination

We identified high proportion of blood cultures taken in the first 72 h without subsequent administration of antibiotics. We assume these occurred due to maternal risk factors, with a possible screening purpose, though our study did not specifically investigate that question. The high rate of blood cultures obtained in infants with respiratory symptoms suggests that respiratory problems do not only trigger blood culture sampling but also facilitates initiation of antibiotic use in early life. While the contamination rate was not extremely high, we hypothesize that false positive blood cultures may be drivers for unnecessary prolonged antibiotic courses. Training to improve sampling technique and avoidance of obtaining blood cultures if low suspicion of infection/asymptomatic infants should be considered.

### Strengths and limitations

A key strength of this study is its use of comprehensive, nationwide data covering all Hungarian NICUs over a four-year period. This provides a robust estimate of the national background EOS incidence and antibiotic exposure across diverse neonatal subgroups. Our data might reflect on the EOS incidence and antibiotic burden in other Central or Eastern European countries. Our analysis is based on data from the HNPR and HCSO, a high-quality and consistent dataset. This along with minimal data loss, increases the reliability of the analyses and reduces the risk of selection bias. In addition, several subgroups were analysed separately. The breakdown by different GA groups allows an easier adoption and integration into daily decision-making and serves as strong foundation for development of new protocols. Some limitations should be acknowledged. The study relies on registry and administrative data, which may be prone to information bias and misclassification. We only have detailed data from level II-III NICUs, but we acknowledge that many infants also receive antibiotics in level I NICUs and postnatal wards, therefore we could only calculate the minimum antibiotic burden of the neonatal population in Hungary, which is clearly an underestimation. Clinical details such as exact timing of antibiotic initiation and cessation, laboratory markers and antibiotic susceptibility patterns beyond blood cultures were not available in the dataset. The HPRN data do not include cases of clinical sepsis with negative cultures. Antibiotic burden could only be calculated based on the total number of antibiotic days given during the NICU stay. The number of neonates discharged and readmitted due to EOS is likely negligible in Hungary. Due to national discharge habits neonates stay in hospital for at least 72 h after birth.

### Implications for practice

Our findings highlight the need for harmonized national approach to the diagnosis and management of early-onset sepsis in Hungary. Coordinated national efforts to standardize clinical practice and promote evidence-based antibiotic stewardship are needed to reduce the substantial and potentially avoidable antibiotic exposure observed in this vulnerable population. As a first step a Hungarian EOS guideline could be adapted based on other European countries’ experiences, surveys and well-structured and established guidelines [31]. If implementing the Kaiser Permanente EOS Calculator in Hungary, our Hungarian CP-EOS incidence (0.20/1000) could be used for risk prediction along with risk factors and clinical data.

Our data also raises awareness of increased overall incidence of *E. coli* EOS, still affecting significantly higher number of premature infants. Substantial mortality occurred due to *E. coli* EOS whereas GBS infections did not lead to neonatal deaths during our study period, similar higher mortality for neonatal sepsis with Gram negative bacteria have been observed in other countries [32–34]

Lumbar puncture was performed in only approximately one fifth of infants with culture-confirmed EOS. Similarly low rates of lumbar puncture in EOS, with the consequent underestimation of early neonatal meningitis, have been reported in Australia [35]. Our retrospective data analysis did not allow investigation of the decisions against performing a lumbar puncture in EOS, further research would be needed to get deeper insight to explore the reasons behind.

Finally, we recommend that the HPRN expand routine data collection to include the specific antibiotics prescribed, antimicrobial susceptibility patterns, and standardized definitions for culture-confirmed sepsis, culture-negative (clinical) sepsis, suspected sepsis, and antibiotic prophylaxis. Harmonization of these definitions nationally, and where possible internationally, would improve consistency in clinical practice, facilitate clear communication, and strengthen the quality and comparability of data for surveillance, benchmarking, and future research.

## Conclusion

This study provides the first national estimates of early-onset sepsis incidence in Hungary and highlights substantial opportunities to optimize antibiotic use. These findings provide a foundation for evidence-based policy and practice, supporting harmonized national approaches to diagnosis, antibiotic stewardship, and the management of newborn infants with suspected early-onset sepsis.

## Supplementary Information

Below is the link to the electronic supplementary material.

Supplementary file1 (DOCX 44 KB)

## Data Availability

No datasets were generated or analysed during the current study.
