# Supplementary material for: Early-onset neonatal sepsis and neonatal antibiotic exposure in Hungary a nationwide population-based study
Source: Eur J Pediatr. 2026 Oct 2;185(11):794. doi: 10.1007/s00431-026-07455-8 (PMC13633339; doi:10.1007/s00431-026-07455-8)
Supplement: Supplementary file 1 [file 431_2026_7455_MOESM1_ESM.docx]

**STROBE Statement—checklist of items that should be included in reports of observational studies**

**Adapted checklist adding the suggested STROBE Neonatal Infection (NI) items (*Lancet Infect Dis* 2016; 16: e202–13)**

**Mari J, et al.** **Early-Onset Neonatal Sepsis and Neonatal Antibiotic use in Hungary – A nationwide population-based study**

|  | Item No. | Recommendation | Page  No. | Relevant text from manuscript |
| --- | --- | --- | --- | --- |
| **Title and abstract** | **1** | (*a*) Indicate the study’s design with a commonly used term in the title or the abstract | Page 2 | See abstract |
|  |  | (*b*) Provide in the abstract an informative and balanced summary of what was done and what was found | Page 2 |  |
| Introduction | | | |  |
| **Background/****rationale** | **2** | Explain the scientific background and rationale for the investigation being reported | Page 3 | See Introduction |
| **Objectives** | **3** | State specific objectives, including any pre-specified hypotheses | Page 3 | See Introduction |
| Methods | | | |  |
| **Study design** | **4** | **Present key elements of study design early in the paper** | Page 4 |  |
| STROBE-NI 4.1: clearly state case ascertainment methods (eg, physician diagnosis, clinical algorithm), documenting individual clinical signs used for diagnosis of possible serious bacterial infection. Give microbiological and/or laboratory and/or radiological criteria for other infectious syndromes (eg, meningitis, sepsis, pneumonia). Include indications for clinical investigations (eg, lumbar puncture)  STROBE-NI 4.2: give criteria used to differentiate between new infection episodes and relapses  STROBE-NI 4.3: for facility-based studies, indicate if the study is of community and/or hospital-acquired infections (HAI), defining HAI using an international standard and presenting specific HAI clinical syndromes separately  STROBE-NI 4.4: state whether this is an outbreak study, and if so define an outbreak, with reference to an international standard  STROBE-NI 4.5: describe sampling strategy (eg, clinical indication *vs* routine surveillance) and sampling details (eg, minimum volumes; timing in relation to antimicrobial administration)  STROBE-NI 4.6: describe conventional and/or molecular microbiological methods used, with details (eg, automation, enrichment steps), and the use of controls  STROBE-NI 4.7: list pathogens that are likely to be identified by microbiological methods used  STROBE-NI 4.8: describe antimicrobial susceptibility tests and thresholds used, with reference to an international standard (eg, CLSI or EUCAST) | | | Population, data and definitions, Page 4    Not relevant for this paper  Not relevant for our study  Not relevant for this paper  Methods page 4-5  Methods page 4  Not relevant for this paper  Not relevant for this paper | |
| **Setting** | **5** | **Describe the setting, locations, and relevant dates, including periods of recruitment, exposure, follow-up, and data collection** |  |  |
| STROBE-NI 5.1: describe the study context in terms of incidence of neonatal mortality, stillbirth, and preterm birth  STROBE-NI 5.2: describe the population included (eg, facility births, referrals from home, referrals from another facility)  STROBE-NI 5.3: for community-based studies, describe care-seeking and adherence and time to referral  STROBE-NI 5.4: for facility-based studies, describe obstetric care (basic or comprehensive), including proportion of births by caesarean section. Report annual number of livebirths per facility and state proportion of births in the study area that occur in hospital (*vs* community)  STROBE-NI 5.5: for facility-based studies, indicate if the facility is public or private, and give the number of health-care staff and their training. Indicate the level of neonatal care available (eg, ventilatory support, indwelling catheters) and investigations available (eg, biochemistry, radiology). Report antimicrobial guidelines used for the empiric management of neonatal sepsis  STROBE-NI 5.6: state the laboratory location and capacity to process different sample types, and give quality control and assurance measures in place | | | Page 4  Page 4  Not relevant for this paper  Population-based cohort  All NICUs caring for neonates in Hungary are public  Not relevant for our study | |
| **Participants** | **6** | *Cross-sectional study*—Give the eligibility criteria, and the sources and methods of selection of participants | Not relevant for this paper |  |
|  |  |  |  |  |
| **Variables** | **7** | Clearly define all outcomes, exposures, predictors, potential confounders, and effect modifiers. Give diagnostic criteria, if applicable | Page 4 |  |
| STROBE-NI 7.1: state criteria used to define clinically significant organisms for each sample type | | | Page 4 | |
| **Data sources/** **measurement** | **8******* | For each variable of interest, give sources of data and details of methods of assessment (measurement). Describe comparability of assessment methods if there is more than one group | Page 6-9 |  |
| **Bias** | **9** | Describe any efforts to address potential sources of bias | Page 5, 8-9 | See statistical methods and limitations |
| **Study size** | **10** | Explain how the study size was arrived at | Registry data |  |

| **Quantitative** **variables** | **11** | Explain how quantitative variables were handled in the analyses. If applicable, describe which groupings were chosen and why | Page 4-5 |  |
| --- | --- | --- | --- | --- |
| **Statistical** **methods** | **12** | (*a*) Describe all statistical methods, including those used to control for confounding | Page 4-5 | See Statistics |
|  |  | (*b*) Describe any methods used to examine subgroups and interactions | Page 4-5 | See Statistics |
|  |  | (*c*) Explain how missing data were addressed | Table 1 | |
|  |  |  |  |  |
|  |  | (*e*) Describe any sensitivity analyses | Not relevant for this paper |  |

| Results | | | | |
| --- | --- | --- | --- | --- |
| **Participants** | **13******* | (a) Report numbers of individuals at each stage of study—eg numbers potentially eligible, examined for eligibility, confirmed eligible, included in the study, completing follow-up, and analysed | Page 4-5, Table 1 |  |
|  |  | (b) Give reasons for non-participation at each stage | Not relevant for this paper |  |
|  |  | (c) Consider use of a flow diagram | Not relevant for this paper |  |
| STROBE-NI 13.1: see figure 2 for suggested components of a flow diagram for neonatal infections | | |  |  |
| **Descriptive** **data** | **14******* | (a) Give characteristics of study participants (eg demographic, clinical, social) and information on exposures and potential confounders | Results/Demographics and Table 1 |  |
|  |  | (b) Indicate number of participants with missing data for each variable of interest | Table 1 |  |
|  |  | (c) *Cohort study*—Summarise follow-up time (eg, average and total amount) | Not relevant for this paper |  |
| STROBE-NI 14.1: describe maternal infections (clinical or on screening—eg, group B streptococcus or HIV) or risk factors for infection (eg, premature rupture of membranes, peripartum fever)  STROBE-NI 14.2: describe key neonatal characteristics, including sex, postnatal and gestational age categories (range and median), birthweight categories (range and median), birth place, feeding (breastmilk or other), and comorbidities  STROBE-NI 14.3: report data on occurrence of individual signs, according to case definitions  STROBE-NI 14.4: give proportion of mothers and neonates with peripartum antibiotic exposure (with or without preadmission exposure for neonates). Report details of antimicrobial drugs (or supportive care) given during the study | | | Not relevant for this paper  Table I  Not relevant for this paper  Not relevant for this paper | |
|  |  |  |  |  |
| **Outcome data** | **15******* | *Cohort study*—Report numbers of outcome events or summary measures over time | Not relevant for our paper |  |
|  |  | *Case-control study—*Report numbers in each exposure category, or summary measures of exposure | Not relevant for this paper |  |
|  |  | *Cross-sectional study—*Report numbers of outcome events or summary measures | Not relevant for this paper |  |
| STROBE-NI 15.1: report the number (and the proportion) of samples microbiologically tested (including lumbar punctures for meningitis cases); the number (and the proportion) that were positive (including thresholds for detection, where applicable); all isolates obtained (including clinically significant and non-significant); and antimicrobial susceptibilities of pathogens, where done  STROBE-NI 15.2: report the number (and the proportion) of babies with microbiologically proven infection (and number of infections per baby), and include this in the flow chart (see figure 2)  STROBE-NI 15.3: report infections by day, for days 0–6. State age categories, if used, defining early-onset and late-onset infection (eg, <72 h and ≥72 h, respectively)  STROBE-NI 15.4: report deaths and any subanalyses by risk groups | | | Not relevant for this paper  Not relevant for this paper  Page 4 See definitions and  outcomes under methods  Table 2 | |
| **Main results** | **16** | (*a*) Give unadjusted estimates and, if applicable, confounder-adjusted estimates and their precision (eg, 95% confidence interval). Make clear which confounders were adjusted for and why they were included | Not relevant for this paper |  |
|  |  | (*b*) Report category boundaries when continuous variables were categorized | Not relevant for this paper |  |
|  |  | (*c*) If relevant, consider translating estimates of relative risk into absolute risk for a meaningful time period | Not relevant |  |

STROBE-NI 16.1: for incidence, give risk per 1000 livebirths, or if alternative denominator used (eg, total births or bed days),

define this clearly	Table 2

| **Other analyses** | **17** | Report other analyses done—eg analyses of subgroups and interactions, and sensitivity analyses | Not relevant for this paper |  |
| --- | --- | --- | --- | --- |

| Discussion | | | | |
| --- | --- | --- | --- | --- |
| **Key results** | **18** | Summarise key results with reference to study objectives | Page 10-12 |  |
| **Limitations** | **19** | Discuss limitations of the study, taking into account sources of potential bias or imprecision. Discuss both direction and magnitude of any potential bias | Page 15-16 |  |
| STROBE-NI 19.1: discuss sources of recruitment bias, particularly regarding the period of time shortly after birth.  State source of denominator data and discuss possible related biases | | | Not relevant for this study | |
| **Interpretation** | **20** | Give a cautious overall interpretation of results considering objectives, limitations, multiplicity of analyses, results from similar studies, and other relevant evidence | Page 11-16 | See Discussion |
| **Generalisability** | **21** | Discuss the generalisability (external validity) of the study results | Page 11-16 |  |
| Other information | |  | | |
| **Funding** | **22** | Give the source of funding and the role of the funders for the present study and, if applicable, for the original study on which the present article is based | Page 18 |  |

*Give information separately for cases and controls in case-control studies and, if applicable, for exposed and unexposed groups in cohort and cross-sectional studies.

**Note:** An Explanation and Elaboration article discusses each checklist item and gives methodological background and published examples of transparent reporting. The STROBE checklist is best used in conjunction with this article (freely available on the Web sites of PLoS Medicine at http://www.plosmedicine.org/, Annals of Internal Medicine at http://www.annals.org/, and Epidemiology at http://www.epidem.com/). Information on the STROBE Initiative is available at www.strobe-statement.org.
